# Exaggerated Immune Reaction to Trichophyton Fungus Results in an Inflammatory Tinea Pedis

**DOI:** 10.26502/aimr.0040

**Published:** 2020-08-21

**Authors:** Robert R Hoopes, Jeffrey B Travers

**Affiliations:** Department of Dermatology, Wright State University Boonshoft School of Medicine, Ohio, United States

**Keywords:** Trichophyton Fungus, Tinea Pedis

## 1. Introduction

Inflammatory skin conditions involving the hands and feet can be the result of many underlying processes. The variety of causal agents can make treatment challenging in determining what approach is best suited for the patient’s underlying diagnosis. Trichophyton is a dermatophyte fungus type that is the cause of many cutaneous fungal infections [1, 2].

Treatment of such infections is usually administered by way of topical antifungal creams [3-5]. We report a case of a patient with a long-standing dermatitis-like reaction on his feet that was found to be positive for the common fungus (Trichophyton) which causes tinea pedis (athlete’s foot).

## 2. Case Report

**Figures 1 and 2:**
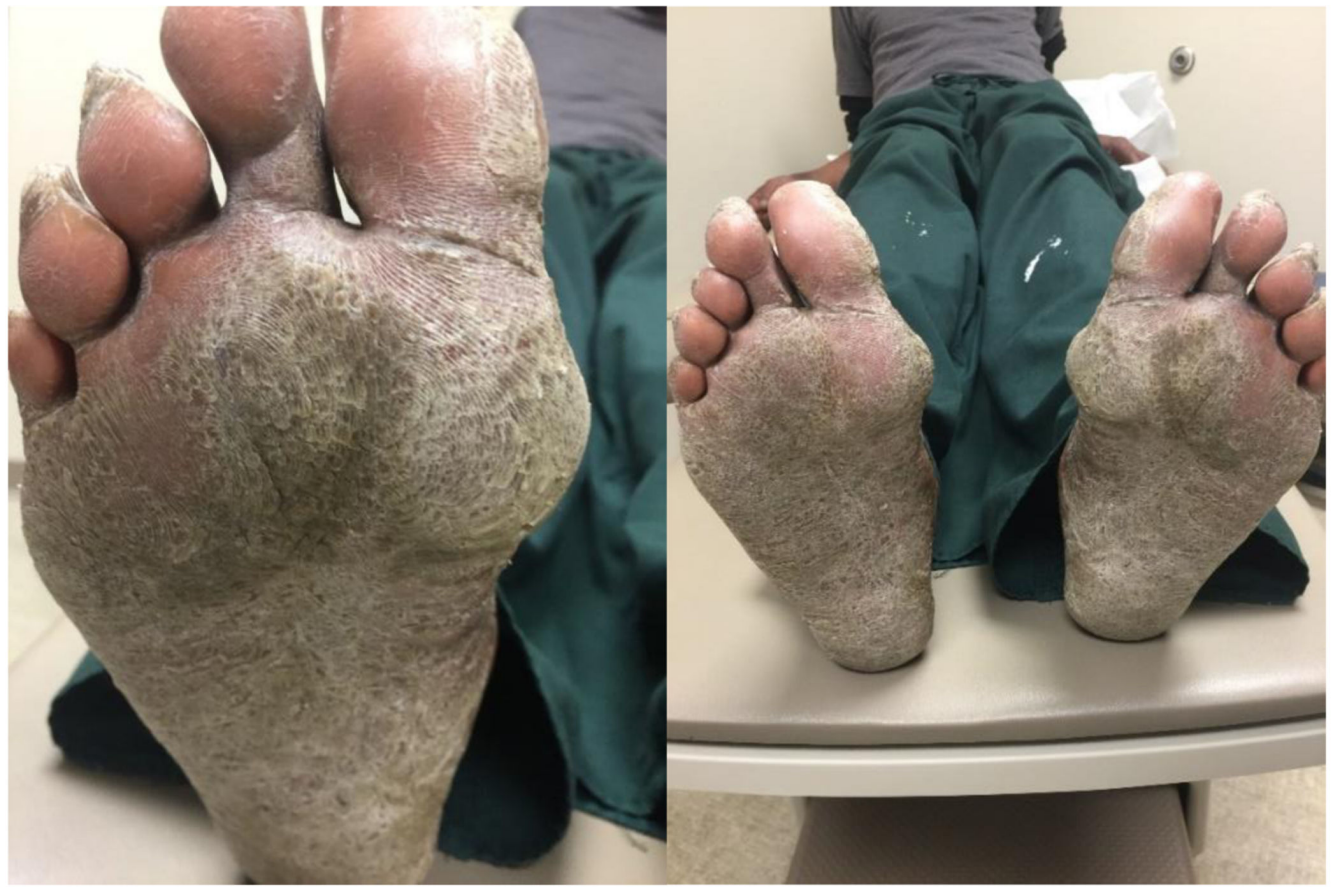
Patient’s right and left plantar surfaces prior to ketoconazole and 20% Urea topical creams.

**Figures 3 and 4:**
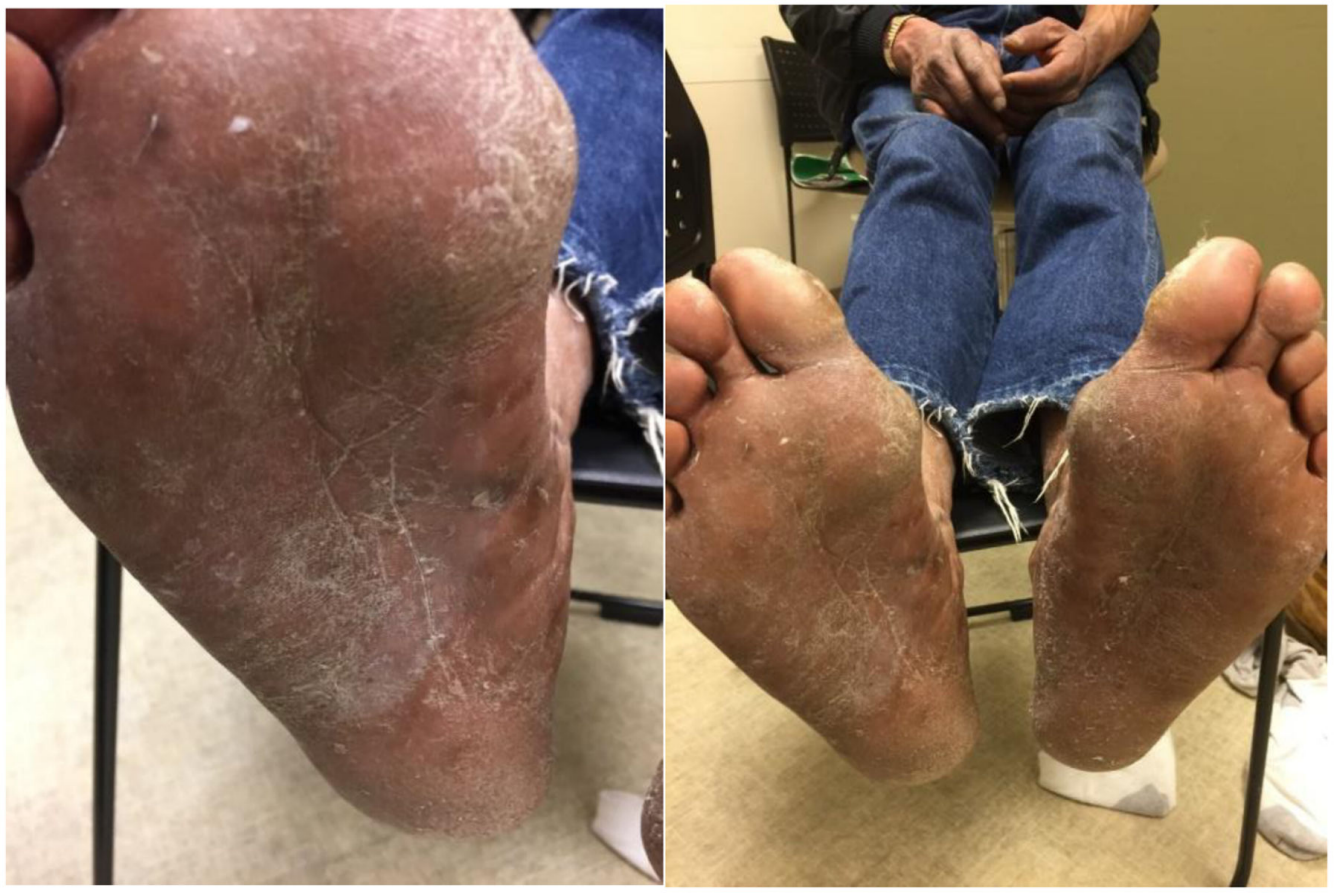
Patient’s right and left plantar surfaces post treatment.

**Figures 5 and 6:**
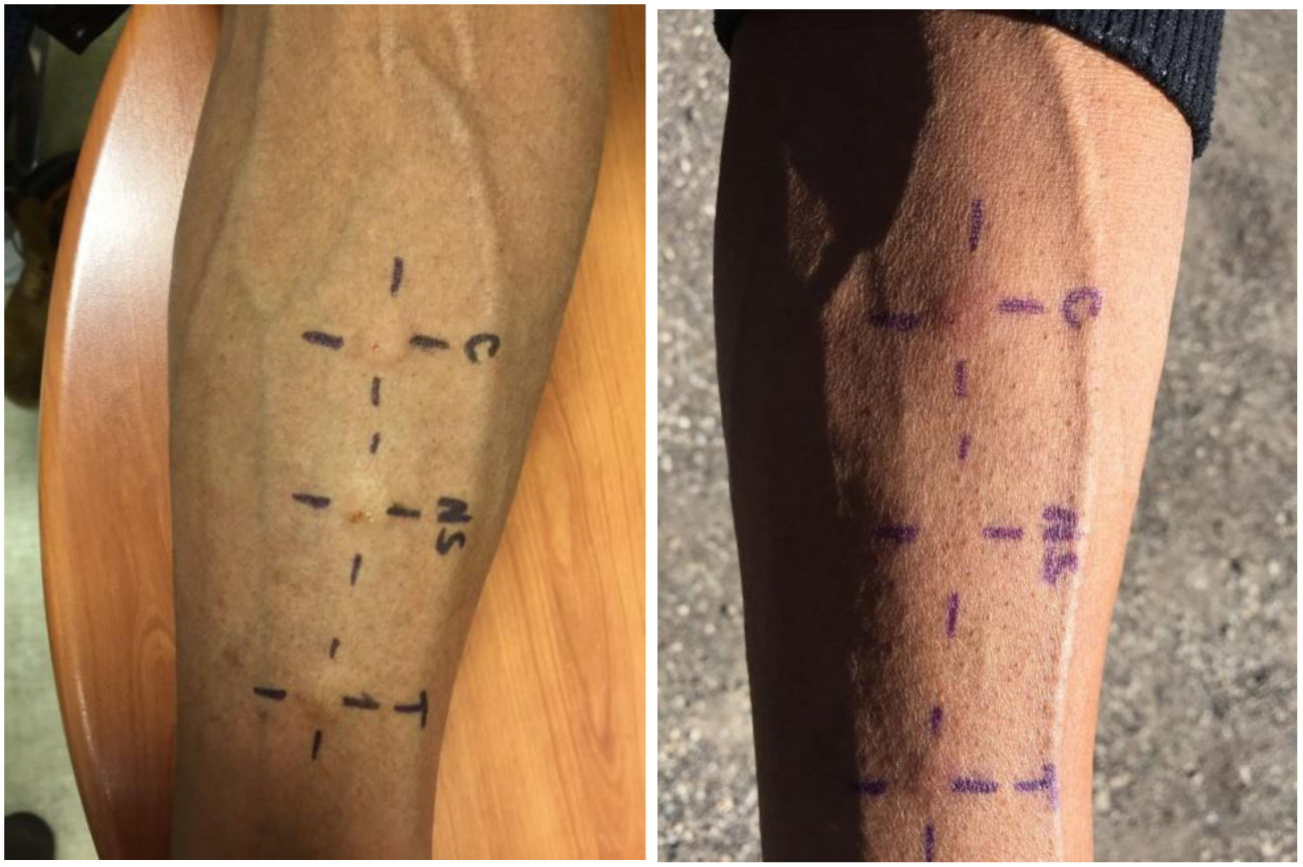
Patient’s left volar forearm at time 0 and 48 hours of allergen application.

We report a case of a 58-year-old African American male with a long history of scaly itchy feet. The patient presented to the clinic on February 8^th^, 2018 with an apparent inflammatory reaction on the plantar surface of both feet (Figures 1 and 2). An initial specimen collection from the patient’s plantar surface revealed hyphae indicative of a fungal infection under KOH preparation. This supported the diagnosis of a tinea pedis infection. The patient was treated with ketoconazole topical cream and 20% Urea creams to be applied daily. Follow-up approximately 6 weeks later revealed resolution of underlying fungal infection with minimal remaining inflammation (Figures 3 and 4). With consideration to the subject’s initial presentation of a possible exaggerated immune reaction caused by Trichophyton, we suspected an underlying allergic response (delayed-type hypersensitivity reaction) to the fungus, instead of a cutaneous fungal infection alone. In order to test this hypothesis, we subjected the patient to intradermal skin testing with intradermal Candida and Trichophyton allergens. On March 21, 2018, the patient underwent application of Candida, normal saline, and Trichophyton allergens on the left volar forearm (Figures 5 and 6).

## 3. Discussion

Assessment at 48 hours revealed a Candida 3+ reaction with an induration of 11.5 x 10.8 mm and a Trichophyton 3+ reaction with an induration of 8.5 x 9.2 mm. Based on previous studies conducted using fungal allergens, it is rare to have a 3+ reaction to Trichophyton. Topical treatment with Ketoconazole and 20% Urea creams resulted in complete resolution as noted in Figures 3 and 4 which further supports the likelihood that this subject was hypersensitive to Trichophyton. We present this case to demonstrate that some patients may present with a pedal inflammatory dermatitis that may be due to an underlying hypersensitivity to Trichophyton resulting in an exaggerated tinea pedis.
